# Reconstruction of fetal brain MRI with intensity matching and complete outlier removal

**DOI:** 10.1016/j.media.2012.07.004

**Published:** 2012-12

**Authors:** Maria Kuklisova-Murgasova, Gerardine Quaghebeur, Mary A. Rutherford, Joseph V. Hajnal, Julia A. Schnabel

**Affiliations:** a Institute of Biomedical Engineering, Department of Engineering Science, University of Oxford, UK; b Neuroradiology, West Wing, Level 1, John Radcliffe Hospital, Oxford OX3 9DU, UK; c Imaging Sciences Department, MRC Clinical Sciences Centre, Imperial College London, Hammersmith Hospital, London W12 0NN, UK

**Keywords:** Fetal MRI, 3D reconstruction, Super-resolution, Bias field, Intensity matching

## Abstract

We propose a method for the reconstruction of volumetric fetal MRI from 2D slices, comprising super-resolution reconstruction of the volume interleaved with slice-to-volume registration to correct for the motion. The method incorporates novel intensity matching of acquired 2D slices and robust statistics which completely excludes identified misregistered or corrupted voxels and slices. The reconstruction method is applied to motion-corrupted data simulated from MRI of a preterm neonate, as well as 10 clinically acquired thick-slice fetal MRI scans and three scan-sequence optimized thin-slice fetal datasets. The proposed method produced high quality reconstruction results from all the datasets to which it was applied. Quantitative analysis performed on simulated and clinical data shows that both intensity matching and robust statistics result in statistically significant improvement of super-resolution reconstruction. The proposed novel EM-based robust statistics also improves the reconstruction when compared to previously proposed Huber robust statistics. The best results are obtained when thin-slice data and the correct approximation of the point spread function is used. This paper addresses the need for a comprehensive reconstruction algorithm of 3D fetal MRI, so far lacking in the scientific literature.

## Graphical abstract

**Graphical abstract:**

**Figure:**
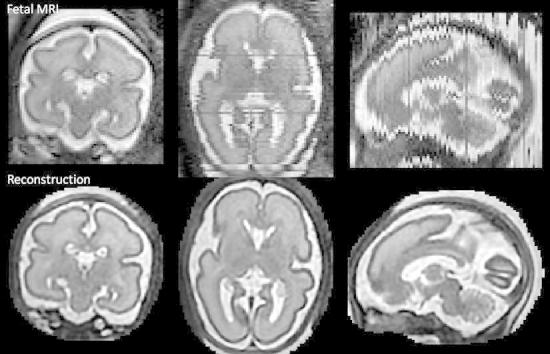

**Highlights:**

► A new method for reconstruction of 3D fetal brain MRI from 2D slices is proposed. ► The super-resolution reconstruction is interleaved with motion correction. ► Corrupted or misaligned slices are automatically excluded. ► Novel intensity matching is shown to be essential for quality of reconstruction. ► Excellent results for clinical and optimized data.

## 1 Introduction

Magnetic resonance imaging (MRI) of the fetal brain has received considerable attention in recent years due to its application in assessing fetal brain development. Clinically, it is used for qualitative assessment of fetal brain abnormalities. Its potential applications include detailed characterization of fetal brain development and identification of deviations in brain growth related to conditions such as intra-uterine growth restriction (IUGR) or preterm birth. Development of new quantitative biomarkers will facilitate better understanding of brain development and subsequent improved management of high-risk pregnancies (Rutherford et al., 2008; Limperopoulos and Clouchoux, 2009).

Due to the long acquisition times of MRI, fetal motion presents a major challenge as 3D MR scanning sequences are not applicable in the presence of unpredictable and fast fetal motion. This has been addressed by the development of ultrafast sequences which are designed to acquire 2D slices and thus freeze the motion in time. The most common sequence used in clinical practice is single-shot fast spin echo (SSFSE) T2-weighted imaging with an acquisition time of 1 s per slice (Prayer et al., 2004). Stacks of thick slices are acquired in three orthogonal directions. The slice thickness is usually 3–4 mm to obtain good contrast-to-noise ratio. Such images provide excellent delineation of fetal brain anatomy and are suitable for qualitative assessment of brain abnormalities. However, compromised fetal brain development, e.g. during IUGR, often manifests as more subtle changes, which can only be assessed by detailed quantitative analysis. Studies on pre-term infants suggest that quantitative biomarkers, such as reduced cortical volume and sulcation index (Tolsa et al., 2004; Dubois et al., 2008) can be used to characterize IUGR. Quantitative analysis of brain development requires volumetric data and very recently, the development of methods for reconstruction of volumetric fetal brain MRI enabled the emergence of quantitative studies for fetal brain development (Damodaram et al., 2009a,b; Gholipour et al., 2011; Clouchoux et al., 2012; Rajagopalan et al., 2011a,b; Habas et al., 2012).

In recent years, methods have been proposed to reconstruct a fetal brain volume from stacks of 2D slices (Studholme, 2011). Such reconstruction presents a number of challenges. Slices need to be automatically aligned to correct for the motion between acquisition of individual slices. Slices with motion artifacts need to be automatically excluded, and inconsistencies in intensity patterns resulting from the motion or acquisition settings, such as variable scaling of the slices and differential bias fields, need to be estimated and corrected for. Finally, the volume has to be reconstructed from irregularly sampled data.

In the first approach proposed by Rousseau et al. (2006), the reconstruction using a weighted sum of Gaussian kernels which represent the point spread function (PSF) is interleaved with slice-to-volume registration and is applied to clinically acquired thick-slice data. If an interpolation approach is used to reconstruct a volume from thick slices, undesirable blurring is introduced into the reconstructed volume. Jiang et al. (2007) propose to acquire many thin slices (1 mm slice thickness) and use multilevel B-splines (Lee et al., 1997) to reconstruct the volume. One advantage of their approach is that the spline interpolation can be tuned to avoid blurring and the use of thin slices enhances this by achieving more isotropic resolution. As the region of interest is oversampled, averaging during reconstruction results in a significant reduction of noise, thus improving the signal-to-noise ratio. Additionally, thin slices can be aligned with significantly reduced target registration error (0.2 mm in simulated experiments). The method was further extended by a model of the motion of the fetus during acquisition to improve the robustness of the registration (Bertelsen et al., 2009). Kim et al. (2010) proposed an alternative motion correction approach, which replaces slice-to-volume registration with a multi-slice registration method constrained by a model of fetal motion. This removes the need for the computationally expensive reconstruction step during alignment of the slices. A modified Gaussian weighted reconstruction is proposed to reduce the blurring effect of thick slices. More recently, super-resolution methods (Milanfar, 2007; Greenspan, 2009) in combination with slice-to-volume registration have been proposed to reduce the blurring effects during reconstruction of thick-slice data (Gholipour and Warfield, 2009; Gholipour et al., 2010). During super-resolution reconstruction, the volume has to be regularized to prevent amplification of noise and registration error, as well as to fill the undersampled regions. To reduce the smoothing effects of regularization, adaptive regularisation techniques can be employed (Peyre, 2011). Rousseau et al. (2010, 2011) proposed to extend super-resolution reconstruction through total variation regularization or an edge-preserving regularization technique developed by Charbonnier et al. (1997). Kim (2011b) proposed an alternative non-iterative solution to selectively weight the Gaussian kernels to obtain a high-resolution volume.

Early reconstruction approaches relied on manual exclusion of the motion corrupted slices. In more recent work, Kim et al. (2010) implemented a rejection of motion corrupted and misregistered slices based on an abnormally high mean squared intensity difference, but in several cases slices had to be manually marked for removal after processing. Gholipour et al. (2010) incorporated robust statistics based on the Huber function into super-resolution reconstruction to automatically reduce the weight of corrupted and misaligned voxels and slices. Recently, Kim et al. (2011a) showed that correction of bias field inconsistencies can improve the quality of the reconstructed volume, which is especially important for correct delineation of fetal lamination.

In this paper we propose a comprehensive method for reconstruction of fetal brain MRI which builds and expands on previous super-resolution approaches (Gholipour and Warfield, 2009; Gholipour et al., 2010; Rousseau et al., 2010). For the first time, we unify all the necessary elements within a common framework to perform simultaneous super-resolution reconstruction, robust statistics and intensity matching to achieve high quality reconstruction. Our contribution compared to previous works is threefold: first, we propose to estimate the differential bias fields and slice-dependent scaling factors during super-resolution reconstruction; second, a novel robust statistics based on an EM framework is proposed to remove artifacts caused by motion-corrupted and misaligned data. Unlike the previously proposed work by Gholipour et al. (2010), the identified outliers are removed completely, thus further reducing the artifacts in the image. Third, we combine the robust super-resolution reconstruction with edge-preserving regularisation Charbonnier et al. (1997) which reduces blurring in the reconstructed images. The reconstruction is interleaved with slice-to-volume registration. We use normalized mutual information as a similarity measure due to its robustness to intensity artifacts and thus remove the need for a model of motion to achieve good quality alignment. Our method is evaluated using simulation of thick slice acquisition for volumetric neonatal data. We also propose a novel leave-one-out analysis to evaluate reconstruction results on real fetal data. Our results show that both intensity matching and the new robust statistics result in a statistically significant improvement in reconstruction performance in simulated and real data experiments. The method produces excellent results for both clinical thick slice data and optimized thin slice data.

## 2 Methods

### 2.1 Slice acquisition model

**Fig. 1:**
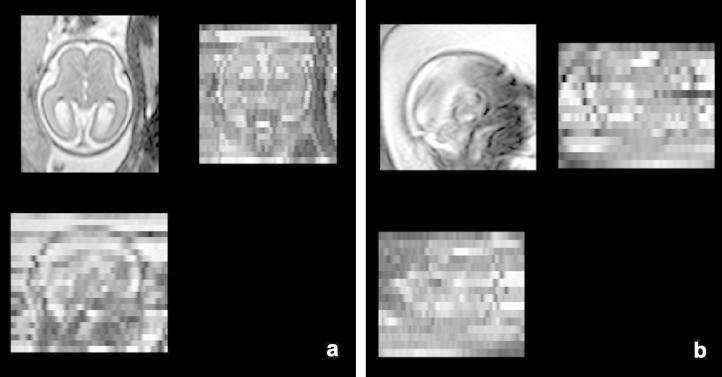
Motion artifacts in fetal MRI shown in three orthogonal plane views. (a) A stack acquired in axial direction exhibiting a smaller amount of motion. The individual images are of high quality but do not form a consistent representation. Structures can still be recognized in the through-plane direction. (b) A stack acquired in sagittal direction with heavy motion. Some slices are corrupted by motion artifacts. Structures cannot be recognized in the through-plane direction.

During acquisition of fetal brain MRI, the fetus is moving freely. The head and brain of the fetus do not deform and so undergo rigid body motion. Several stacks of 2D slices are acquired in different orientations, one slice at a time. If the motion of the fetal head is relatively slow compared to the slice acquisition time, the slices represent accurate 2D images of the fetal head, while the movement can be observed between the slices. Let us denote the acquired slices by $Y_{1}\text{,}\ldots \text{,}Y_{K}$ and voxels of the *k*th slice by $y_{\mathrm{jk}}\text{,}\;j=1\text{,}\ldots \text{,}N_{k}$. We aim to find an unknown volume *X*, consisting of voxels $x_{1}\text{,}\ldots \text{,}x_{N}$, which represent an accurate 3D image of the fetal head. The spatial relationship between voxels of the acquired slices and the reconstructed volume is represented by matrices $M_{k}$, with each row $\left\{m_{\mathrm{ij}}^{k}\text{,}i=1\text{,}\ldots \text{,}N\right\}$ describing the spatially aligned discretized point spread function (PSF) for the acquisition of a voxel $y_{\mathrm{jk}}$ from volume *X*. The PSF is determined by the acquisition and hence is known, but the positions and orientations of the PSF kernels, determined by the spatial positions of the slices, have to be estimated. Even though fast acquisition of a single 2D slice freezes motion in time in most cases, a number of slices may exhibit artifacts due to the sudden motion of the fetus, especially for younger fetuses. An example of motion artifacts is shown in Fig. 1. These corrupted slices have to be identified and excluded from the reconstruction. Movement of the fetus also means that the position of the fetus relative to the scanner is changing. Thus the inhomogeneity of the magnetic field affects the intensities of slices according to their position in the scanner, rather than their position in the fetal head, creating inconsistencies in the reconstructed volume. Similarly, individual slices are affected by different scaling factors. Our model therefore assumes slice-dependent bias fields $B_{k}=\{b_{\mathrm{jk}}\text{;}\;j=1\text{,}\ldots \text{,}N_{k}\}$ and scaling factors $s_{k}$, which have to be estimated during reconstruction. We denote scaled and bias corrected slices by $Y_{k}^{*}$, and intensities of their voxels by $y_{\mathrm{jk}}^{*}$. The relationship between acquired slices and the unknown volume, or the slice acquisition model, can thus be expressed as

(1) $$Y_{k}^{*}=M_{k}X\text{;}\;y_{\mathrm{jk}}^{*}=s_{k}\exp(-b_{\mathrm{jk}})y_{\mathrm{jk}}$$

In many approaches, a multiplicative bias field model is transformed to an additive one by logarithmic transformation of the image intensities (Wells et al., 1996; Leemput et al., 1999). To avoid the logarithmic transformation, we work directly with the multiplicative bias field. For this, we use multiplicative the exponential model proposed by Ashburner and Friston (2005) which ensures that this bias field model is equivalent to the one proposed in works which use a logarithmic transformation of intensities.

### 2.2 Point spread function

A good approximation of the PSF is essential so that high quality reconstruction is obtained by solving Eq. 1. The exact shape of the PSF is acquisition dependent, and Jiang et al. (2007) described the PSF for ssFSE sequences. The in-plane PSF of the acquired slice can be generally considered to be a sinc function with full width at half maximum (FWHM) of its central peak equal to 1.2 × in-plane resolution. The PSF in through-plane direction is the slice profile. Jiang et al. (2007) measured the slice profile of the ssFSE sequence and found it to be approximately Gaussian, with FWHM equal to the slice thickness. Therefore in this paper we treat the PSF as a 3D Gaussian function with FWHM equal to the slice-thickness in the through-plane direction and 1.2 × voxel size in-plane (an approximation of the sinc function).

### 2.3 Super-resolution reconstruction of a 3D volume from thick slices

Standard imaging protocols for fetal brain MRI consist of several stacks of thick slices, to achieve fast acquisition and good signal-to-noise ratio. During acquisition of thick slices, weighted averaging of the MR signal is occurring in through-plane direction. If the intensities of thick slices are only interpolated to recover a 3D volume, blurring is introduced into the reconstructed image, decreasing the image definition. Knowledge of the slice acquisition model can be used to recover a higher resolution 3D volume from multiple thick slices (Gholipour et al., 2010; Rousseau et al., 2010). The intensity corrected slice can be simulated from the reconstructed volume *X* using Eq. (1). If the spatial alignment, PSF, bias fields and scaling factors are all known, the volume *X* can be recovered by minimizing the sum of squared differences of errors $e_{\mathrm{jk}}$ between voxels of intensity corrected acquired slices and simulated slices:

(2) $$e_{\mathrm{jk}}=y_{\mathrm{jk}}^{*}-\underset{i}{\sum }m_{\mathrm{ij}}^{k}x_{i}$$

Because of the instability of such reconstruction, regularization of the reconstructed image *X* is necessary (Charbonnier et al., 1997; Rousseau et al., 2010; Gholipour et al., 2010). To minimize the smoothing effects of regularization on image features, edge-preserving regularization can be used (Charbonnier et al., 1997; Rousseau et al., 2010). In this paper we follow the approach of Charbonnier et al. (1997), who proposes the regularization term

(3) $$R(X)=\underset{i}{\sum }\underset{d}{\sum }\varphi \left(\frac{x_{i+d}-x_{i}}{\delta |d|}\right)$$

where $\varphi (t)=2\sqrt{1+t^{2}}-2$ and d represents a vector between the index of a voxel and one of its 26 neighbors. (Note that in practice $i\in N^{3}$ and we assume isotropic resolution for the volume *X*.) Parameter δ controls how big the difference between neighboring voxels must be so that it is considered to be an edge. Applying gradient descent to the objective function $\sum_{\mathrm{jk}}e_{\mathrm{jk}}^{2}+\lambda R(X)$ results in an updating equation for volume *X*:

(4) $$x_{i}^{\left(n+1\right)}=x_{i}^{n}+\alpha \underset{\mathrm{jk}}{\sum }m_{\mathrm{ij}}^{k}e_{\mathrm{jk}}^{n}+\alpha \lambda \frac{\partial }{\partial x_{i}}R(X)$$

where $\frac{\partial }{\partial x_{i}}R(X)=\frac{1}{\delta^{2}}\sum_{d}b_{i}^{d}(x_{i+d}^{n}-x_{i}^{n})$. The value $b_{i}^{d}$ determines how much smoothing is performed for voxel *i* in direction d and is calculated as

(5) $$b_{i}^{d}=\frac{1}{|d|\sqrt{1+\frac{x_{i+d}-x_{i}}{\delta |d{|}^{2}}}}\text{.}$$

### 2.4 Motion correction

**Fig. 2:**
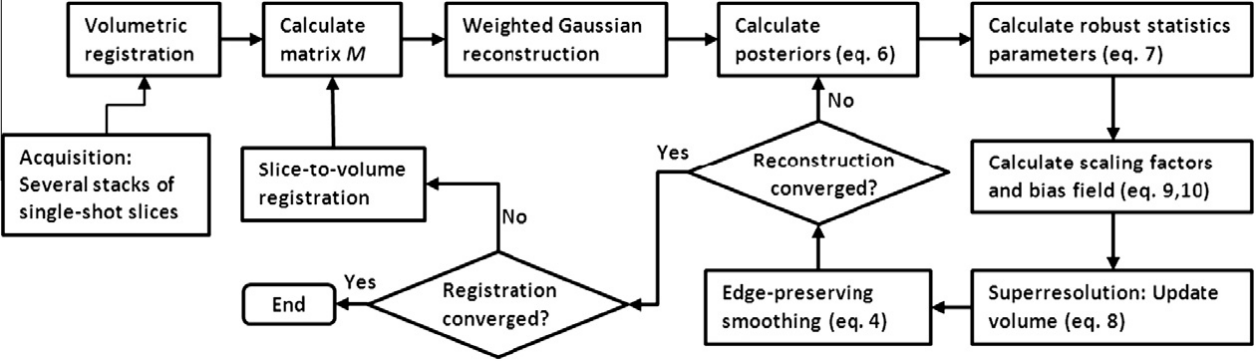
Overview of the proposed methodology.

To correct for motion between slices we use the original scheme proposed by Rousseau et al. (2006) and Jiang et al. (2007). Stacks are first co-aligned using volumetric rigid registration and the first estimate of the volume is reconstructed. Afterwards, each slice is registered to the reconstructed volume separately. Slice-to-volume rigid registration is interleaved with super-resolution reconstruction for a fixed number of iterations, which is determined experimentally. The advantage of this simple scheme is that any existing similarity measure can be used to maximize the quality of the alignment. We chose normalized mutual information (Studholme et al., 1999), as it is independent of the scaling factors and has low sensitivity to low magnitude bias fields, which are typically present in MRI acquired at 1.5 T. An overview of this interleaved scheme is presented in Fig. 2.

### 2.5 Voxel-wise robust statistics

Even though acquisition of 2D MR slices is fast, sudden fetal motion may still result in corruption of some of them. Additionally, due to the limitations of the registration methodology, the slices will be partially misregistered and some may be completely misplaced. In this paper we propose a classification of each slice voxel into two classes – inliers and outliers – within an EM framework. The probability density function (PDF) for the inlier class is modeled as a zero-mean Gaussian distribution $G_{\sigma }(e)$ with variance $\sigma^{2}$, and outliers are modeled by a uniform distribution with constant density *m*, which can be chosen as a reciprocal of the range of values *e*. A mixing proportion *c* represents the proportion of inliers, or correctly matched voxels. The likelihood of observing an error $e_{\mathrm{jk}}$ can then be expressed as

$$P(e_{\mathrm{jk}}|\sigma \text{,}c)=G_{\sigma }(e_{\mathrm{jk}})c+m(1-c)$$

We seek to maximize the log-likelihood $\log P(Y|\Phi )=\sum_{\mathrm{kj}}\log P(e_{\mathrm{jk}}|\sigma \text{,}c)$, where parameters Φ consist of the reconstructed volume *X*, bias field *B*, scaling factors *S*, variance $\sigma^{2}$ of the distribution of errors $e_{\mathrm{jk}}$, and proportion of correctly matched voxels *c*. In this framework, the posterior probability of a voxel being classified as an inlier is

(6) $$p_{\mathrm{jk}}=\frac{G_{\sigma }(e_{\mathrm{jk}})c}{G_{\sigma }(e_{\mathrm{jk}})c+m(1-c)}$$

Parameters σ and *c* are updated using

(7) $$\sigma^{2}=\frac{\sum_{\mathrm{kj}}p_{\mathrm{jk}}e_{\mathrm{jk}}^{2}}{\sum_{\mathrm{kj}}p_{\mathrm{jk}}}\;\text{and}\;c=\frac{\sum_{\mathrm{kj}}p_{\mathrm{jk}}}{\sum_{k}N_{k}}$$

**Fig. 3:**
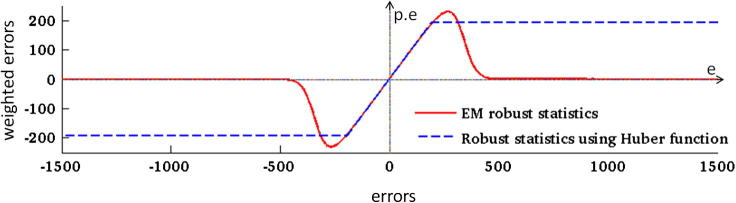
Influence functions of EM and Huber robust statistics. During an iteration of the super-resolution algorithm, errors $e_{\mathrm{jk}}$ are redistributed to update the reconstructed volume (Eq. (4)). If robust statistics are used, values $p_{\mathrm{jk}}e_{\mathrm{jk}}$ are redistributed instead (Eq. (8)). Robust EM statistics transform large errors to values close to zero (red line) while the Huber function only thresholds the error values at a certain value (dashed blue line). The *x*-axis represents error values $e_{\mathrm{jk}}$, and the *y*-axis represents error values after applying robust statistics. (For interpretation of the references to color in this figure legend, the reader is referred to the web version of this article.)

The first term of the objective function for super-resolution reconstruction will change to $\sum_{\mathrm{kj}}p_{\mathrm{jk}}e_{\mathrm{jk}}^{2}$. The updating equation for volume *X* (Eq. (4)) will change to

(8) $$x_{i}^{n+1}=x_{i}^{n}+\alpha \underset{\mathrm{kj}}{\sum }p_{\mathrm{jk}}m_{\mathrm{ij}}^{k}e_{\mathrm{jk}}$$

where the regularization term has been omitted for simplicity but has been included in the method. At each iteration, errors $e_{\mathrm{jk}}$ are calculated and then redistributed to the reconstructed volume *X* according to the PSF. Due to the robust statistics, values $p_{\mathrm{jk}}e_{\mathrm{jk}}$ are redistributed instead of the errors $e_{\mathrm{jk}}$. The advantage of the proposed robust statistics is that these values converge to zero with large errors (see Fig. 3), unlike Huber statistics as used in Gholipour et al. (2010), where errors are instead thresholded at a certain value and thus artifacts cannot be fully removed from the reconstructed volume. The proposed EM robust statistics is in fact a redescending M-estimator with influence function $\frac{e.G_{\sigma }(e)c}{G_{\sigma }(e)c+m(1-c)}$, which is automatically fitted to the data by estimating parameters σ and *c* using the EM algorithm. Nevertheless, the proposed framework is not restricted to EM robust statistics. Using Huber robust statistics results in simply replacing Eq. (6) by $p_{\mathrm{jk}}=1$ if $|e_{\mathrm{jk}}|⩽\gamma$ and $p_{\mathrm{jk}}=\gamma /|e_{\mathrm{jk}}|$ if $|e_{\mathrm{jk}}|>\gamma$. Similarly to Gholipour et al. (2010), we set γ to $1.35*\text{median}(|e_{\mathrm{jk}}|)$.

### 2.6 Slice-dependent robust statistics

In the context of fetal brain MRI, voxel-wise robust statistics cannot sufficiently deal with all types of errors. Brain MRI largely consists of a few tissue classes with approximately constant intensities. Therefore, incorrectly aligned or even corrupted slices may contain a number of voxels which are well matched with the volume *X* in terms of intensity. We found that voxel-wise classification is not sufficient to remove artifacts of motion corruption and misregistration. This was previously reported by Gholipour et al. (2010), who suggest to combine voxel-wise robust statistics with slice-dependent robust statistics using the Huber function. In this paper we also classify slices into inliers and outliers using the EM algorithm. The classification of slices is applied to values $\sqrt{(\sum_{j}p_{\mathrm{jk}}^{2})/N_{k}}$. Slices are classified into inliers and outliers using a mixture of two Gaussians as a model for the PDF. We found that due to the effects of averaging over the voxels of each slice, this PDF is more suitable for classification of slices than a mixture of a Gaussian and a uniform distribution used for voxel-wise statistics, which was presented in Section 2.5. Standard EM estimation of the means, variances and mixture proportions of the inlier and outlier classes (Duda et al., 2001) are used to calculate inlier class slice posteriors $p_{k}^{\mathrm{slice}}$. Consequently, the value $p_{k}^{\mathrm{slice}}p_{\mathrm{jk}}e_{\mathrm{jk}}$ is redistributed to the volume at each super-resolution iteration. This is followed by the edge-preserving smoothing described in Section 2.3. Robust statistics enables us to use a weighted version of this smoothing scheme, with the weights for each voxel in the volume given by $\sum_{\mathrm{kj}}p_{k}^{\mathrm{slice}}p_{\mathrm{jk}}m_{\mathrm{ij}}^{k}$.

### 2.7 Intensity matching and bias correction

Acquired slices coming from the MR scanner are often inconsistently scaled and affected by inconsistent bias fields. The EM framework offers means to recover scales *S* and bias fields *B* during the reconstruction process by minimizing $F(S\text{,}B)=\sum_{\mathrm{jk}}p_{k}^{\mathrm{slice}}p_{\mathrm{jk}}e_{\mathrm{jk}}^{2}$. Setting derivatives of F(S,B) w.r.t scales *S* to zero yields

(9) $$s_{k}=\frac{\sum_{j}p_{\mathrm{jk}}\exp(-b_{\mathrm{jk}})y_{\mathrm{jk}}\sum_{i}m_{\mathrm{ij}}^{k}x_{i}}{\sum_{j}p_{\mathrm{jk}}{\left(\exp(-b_{\mathrm{jk}})y_{\mathrm{jk}}\right)}^{2}}$$

The above equation can be interpreted as the calculation of a scaling factor between weighted averages of intensities of the acquired and simulated slice. The weights are derived from posterior probabilities of belonging to the inlier class $p_{\mathrm{jk}}$ and take the form $p_{\mathrm{jk}}\exp(-b_{\mathrm{jk}})y_{\mathrm{jk}}$. As the scale factors are multiplicative parameters, similar to the bias fields, this result gives us an insight into the behavior of weights in multiplicative models which incorporate EM classification. For calculation of differential bias fields we choose the original approach of Wells et al. (1996) for its computational efficiency. Unlike this well established methodology though, we do not perform a logarithmic transformation of intensities in a pre-processing step to obtain additive bias fields, but assume a multiplicative model. The differential bias fields can be estimated at each iteration by comparing voxels of the acquired slices $y_{\mathrm{jk}}$ to the voxels of simulated slices $\sum_{i}m_{\mathrm{ij}}^{k}x_{i}$. As our model of the bias field is a multiplicative exponential, the differential bias field that is still present in the corrected slice voxels $y_{\mathrm{jk}}^{*}$ at the (n+1)th iteration can be estimated from the residual $r_{\mathrm{jk}}=\log(y_{\mathrm{jk}}^{*}/\sum_{i}m_{\mathrm{ij}}^{k}x_{i})$. If the slices are not perfectly aligned or if they are corrupted by sudden fetal motion, this will be reflected in residuals $r_{\mathrm{jk}}$. To take advantage of the robust statistics, the differential bias field can be obtained by a weighted Gaussian smoothing. For multiplicative models, information about well matched voxels in form of $p_{\mathrm{jk}}$ transforms into weights $w_{\mathrm{jk}}=y_{\mathrm{jk}}^{*}p_{\mathrm{jk}}$. Thus the update equation for bias fields becomes

(10) $$b_{\mathrm{jk}}^{\left(n+1\right)}=b_{\mathrm{jk}}^{n}+\frac{\sum_{l}w_{\mathrm{lk}}^{\left(n+1\right)}G_{\sigma_{B}}(d_{\mathrm{jl}}^{k})r_{\mathrm{lk}}^{\left(n+1\right)}}{\sum_{l}w_{\mathrm{lk}}^{\left(n+1\right)}G_{\sigma_{B}}(d_{\mathrm{jl}}^{k})}$$

where $d_{\mathrm{jl}}^{k}$ represents the spatial distance between voxels *jk* and *lk*, and $\sigma_{B}$ is the standard deviation for the smoothness of the bias field. Note that Eqs. (9) and (10) can converge to a whole subspace of solutions. Scale $s_{k}$ is equivalent to subtracting a constant $\log s_{k}$ from the bias field of slice *k*. Even though the concept of scale is somewhat redundant in the presence of bias field estimation, we prefer to retain it to put additional constraints on the subspace of solutions. We require the product of all scales to be equal to one, and bias fields for all slices to have zero means. This helps to stabilize overall scaling of the image. In our experiments we observed that if such constraints are not set, the intensity range of the reconstructed volume decreases with each iteration and would eventually converge to a zero image. Indeed a reduction of intensity values would decrease the absolute values of the errors $e_{\mathrm{jk}}$ and consequently the value of the objective function. Note that the method does not correct the bias field in the reconstructed image *X*, but only differential biases between the slices. Thus the algorithm can converge to an estimate of a 3D volume corrupted by any smooth bias field.

## 3 Implementation

### 3.1 Data preprocessing

Fetal MR images are acquired as stacks of slices using a standard single-shot fast spin-echo (SSFSE) T2-weighted sequence, with several stacks in each of the three orthogonal directions. During scanning the fetus can move in relation to the mother, and rigid alignment, which is used to correct for motion, cannot account for such changes. Presence of maternal tissue in the image can therefore increase the alignment error in the region of the fetal brain. We therefore create a fetal head mask in all the stacks in the pre-processing step. One of the stacks, typically the one with least motion and artifacts, is chosen as a target. The fetal head is then manually segmented in the target volume. This only takes a couple of minutes due to the large voxel size in the through-plane direction. In case of thin-slice data, the target volume can be first downsampled, then manually brainmasked and upsampled to the original resolution. The mask is then smoothed using a Gaussian filter and thresholded. Other stacks are automatically aligned with the target using the volumetric rigid registration implemented in IRTK.^2^ The registration is initialized with different orientations and the images are aligned using gradient descent. The result with the highest similarity, in this paper normalized mutual information (Studholme et al., 1999), and sufficiently large overlap is chosen to provide an initial alignment of the stacks. The mask is automatically transferred to all other image slices in the stack. All stacks are cropped and padded according to the mask. As the images in our database are sometimes inconsistently scaled, we calculate a scaling factor for each masked stack to set the mean intensity to a pre-defined value. This ensures a good quality initialisation for intensity matching and standardisation of parameter δ for edge-preserving smoothing.

### 3.2 Slice-to-volume registration

The motion correction of individual slices is performed using rigid registration implemented within the IRTK software package. After the volumetric registration of the stacks, the algorithm iterates between reconstruction and slice-to-volume registration for a pre-defined number of iterations. We choose 12–16 iterations for younger fetuses which typically exhibit lots of motion, and 6–9 iterations for older fetuses, for whom the motion is more restricted. To decrease the risk of the algorithm getting trapped in a sub-optimal solution, we use a smoothing scheme, where parameter λ, which determines the amount of smoothness in the reconstructed image, is set large for the first iterations and then is gradually decreased. We experimentally determined suitable values for λ to be $0.16\delta^{2}$ or $0.08\delta^{2}$ for the first motion correction iteration. The smoothing at the final step is mostly determined by the magnitude of the noise in the data, typically $\lambda =0.02\delta^{2}\text{,}0.01\delta^{2}$ or $0.005\delta^{2}$. This temporally adaptive smoothing scheme helps the algorithm to converge faster and improves the accuracy of the final alignment of the slices.

### 3.3 Volumetric reconstruction

At each iteration of motion recovery, the estimate of the volume has to be reconstructed using the latest estimate of the alignment of the slices. The first step is the calculation of matrices $M_{k}$, which consist of positioning and orienting Gaussian kernels according to the transformation between each slice and volume followed by sampling to the grid of the volume. The initial estimate of the volume is calculated using weighted Gaussian reconstruction (Rousseau et al., 2006; Ohbuchi et al., 1992). The slices are then simulated from the volume, and compared to the acquired slices to calculate the errors $e_{\mathrm{jk}}$ (Eq. (2)). Next, the expectation step is performed, where voxel weights $p_{\mathrm{jk}}$ (Eq. (6)) and slice-dependent weights $p_{\mathrm{jk}}^{\mathrm{slice}}$ are calculated. This is followed by the maximization step, in which we calculate robust statistics parameters, namely the variance σ and mixing proportion *c* for voxels-wise statistics (Eq. (7)) and means, variances and mixing proportions for classification of inlier and outlier slices. The maximization step is completed by calculation of scaling factors $s_{k}$ (Eq. (9)) and bias fields $b_{\mathrm{jk}}$ (Eq. (10)). The standard deviation for smoothness of the bias field was experimentally set to 12 mm. Finally, the super-resolution step is performed in which volume *X* is updated according to Eq. (8), followed by the weighted version of edge-preserving smoothing (Section 2.3). During edge-preserving smoothing, the parameter δ determines how much difference in intensity the neighboring voxels have to prevent the smoothing. We set δ approximately to the half of the difference between intensity means of gray matter and white matter. The super-resolution reconstruction is run for a pre-defined number of iterations. We found that the relative error of the objective function converges very slowly due to the super-resolution component, especially if a low weight is given to smoothing. Additionally, the number of iterations is a very stable indicator of the sharpness of the resulting image. We therefore run 10 expectation–maximization (EM) iterations of volume reconstruction during each motion correction iteration, and 30 EM iterations during final reconstruction to obtain high-contrast final volume.

### 3.4 Summary of the reconstruction algorithm

1. Volumetric registration of stacks against template stack.
2. Masking of the head and cropping of all stacks.
3. Intensity matching of the stacks.
4. Motion correction: For each motion-correction iteration.
  (a) If not the first iteration perform slice-to-volume registration.
  (b) Update smoothing parameter α.
  (c) Calculate matrix *M* of discretized oriented PSFs.
  (d) Gaussian weighted reconstruction of the volume.
  (e) Reconstruction of the volume: For each super-resolution iteration.
    i. Estimate robust statistics posteriors (Eq. (8).
    ii. Estimate robust statistics parameters (Eq. (8).
    iii. Estimate the scales and bias fields (Eq. (8).
    iv. Super-resolution: Update volume (Eq. (8).
    v. Edge-preserving smoothing (Eq. (4).)

## 4 Results

Evaluation of the methodology for reconstruction of fetal MRI is a non-trivial task due to the lack of ground truth. It therefore became a standard to simulate acquisition of 2D slices corrupted by motion from neonatal 3D MRI (Rousseau et al., 2006; Jiang et al., 2007; Kim et al., 2010; Gholipour et al., 2010). In this paper we perform such simulation using MRI of a preterm neonate. Additionally, for the first time, we propose to evaluate the reconstruction of the clinical fetal data in a leave-one-out fashion, by excluding one stack and comparing the reconstructed volume to acquired slices in the excluded stack.

### 4.1 Simulated experiment

To simulate the acquisition of fetal brain MRI, we used a T2-weighted fast-spin echo image of a preterm neonate with gestational age (GA) of 27 weeks, acquired at Hammersmith Hospital, London, on a 3T Philips Intera system with MR sequence parameters TR = 8620 ms, TE = 169 ms, and voxel sizes 1 mm × 1 mm × 1 mm. The image was acquired as several stacks of 2D slices, with slice thickness 2 mm and slice overlap 1 mm. The in-plane resolution was 1 mm. The volume was reconstructed using the method proposed by Jiang et al. (2007). Though this image was acquired with a sequence similar to fetal sequences, the neonate was still during acquisition and motion artifacts were therefore negligible. Additionally, as the neonate is asleep during scanning, longer acquisition times are possible, enabling dense sampling of the neonatal head. Thus the quality of reconstructed neonatal MRI acquired this way is comparable to 3D neonatal MRI, with the advantage that the occasional motion artifacts can be corrected for.

**Fig. 4:**
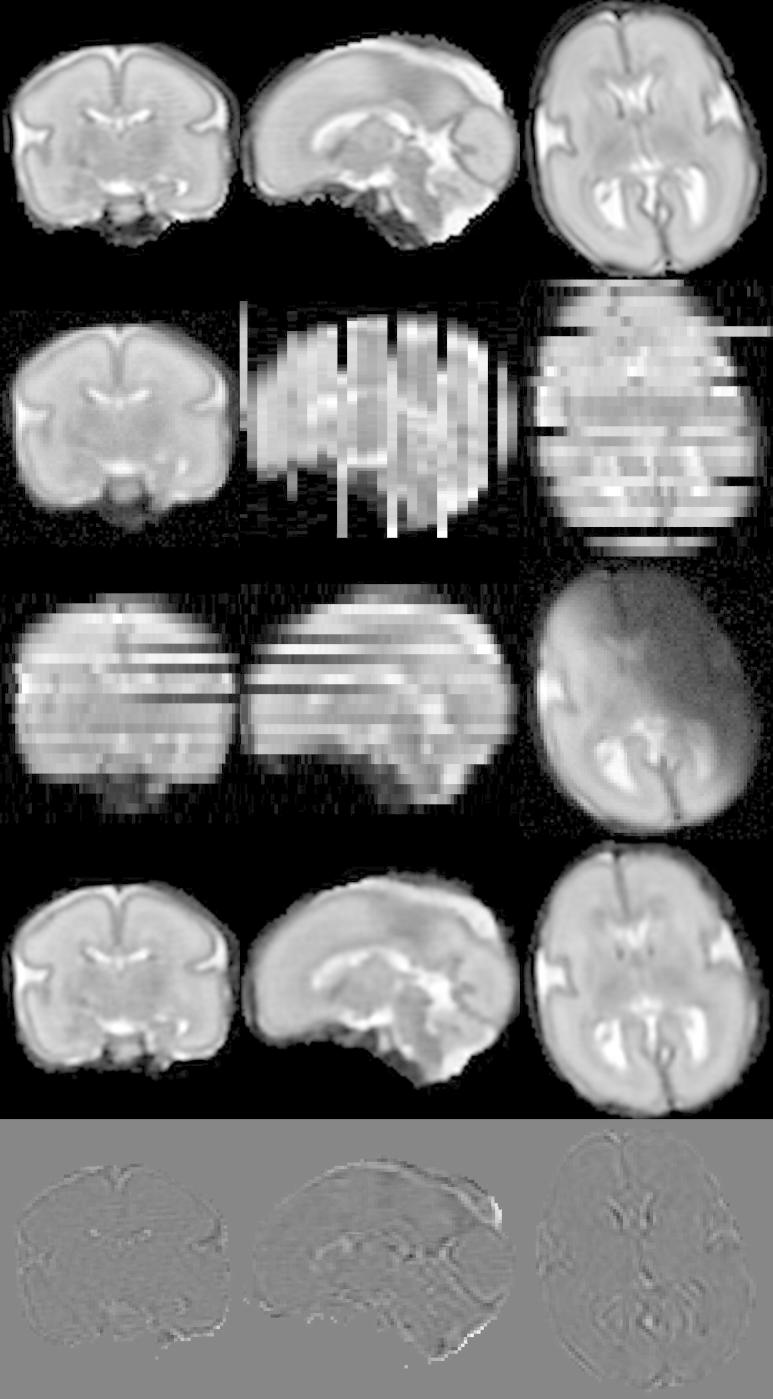
Simulation of fetal brain MRI from a neonatal subject with GA 27 weeks. First row: Neonatal volume. Second row: Simulated coronal stack with 6 slices with large displacement to simulate misregistered outliers. Third row: Simulated transversal stack with three corrupted slices. Fourth row: Reconstruction using six stacks, which include stacks shown in the second and third row, demonstrates the good performance of the method when compared to neonatal volume int the first row. Fifth row: The difference between original and reconstructed image.

The neonatal image is shown in the first row of Fig. 4. From this volume $X^{*}$, the stacks of 2D slices with a slice thickness of 3 mm and in-plane resolution 1 mm×1mm were simulated in three orthogonal directions, 26 slices per stack. First, the motion of the slices was randomly generated. The resulting transformations were then used to calculate the matrices $M_{k}$ defined in Eq. (1) and voxels $y_{\mathrm{jk}}$ of slices $Y_{k}$ were simulated as $\sum_{i}m_{\mathrm{ij}}^{k}x_{i}^{*}$. We added Gaussian noise with σ=0.025μ(μ denotes image mean) to the simulated slices, as well as random bias fields which have been smoothed with a Gaussian filter using σ=12mm. Additionally, slices were multiplied by random scaling factors in the range [0.8, 1.2]. Six slices per three orthogonal stacks were assigned displacements large enough so that the registration algorithm is unable to recover the correct position of the slice (see Fig. 4, second row). This was done to test the capability of the method to robustly exclude misaligned slices. We also added motion corruption to five slices per three orthogonal stacks (see Fig. 4, third row). We simulated three sets of three stack simulations and three sets of six stack simulations, see Table 1. The fourth row of Fig. 4 shows the volume reconstructed from six stacks using the proposed method.

To perform a quantitative evaluation, the simulated stacks were used for reconstruction of a volumetric high-resolution image using four different methods: (1) the full method proposed in this paper, (2) the method with Huber robust statistics and intensity matching proposed in this paper, (3) the method without robust statistics and with intensity matching proposed here, (4) the method with EM robust statistics proposed here, but without intensity matching. To establish the capability of the proposed method to recover correct intensity matching, we also reconstructed the image from the stacks which have not been corrupted by any bias fields and scaling factors (reference method). No intensity matching was applied. The summary of the five methods is shown in Table 2.

We compared the reconstruction results with the original data in two different ways. Firstly, the reconstructed images were directly compared to the original neonatal image used for simulation. The reconstructed images were first rigidly aligned with the original volume and resampled. Subsequently, the reconstructed image was scaled and bias corrected to match the original volume, using the intensity matching method developed in this paper (Eqs. 6, 7, 9, 10). The intensity error between the reconstructed image and the original image was then calculated as the normalized root mean squared error (NRMSE), defined as root mean squared error divided by the average intensity of the original volume:

(11) $$\mathrm{NRMSE}(X^{\prime }\text{,}X^{*})=\frac{\sqrt{\sum_{i}{\left(x_{i}^{\prime }-x_{i}^{*}\right)}^{2}/N}}{\sum_{i}x_{i}^{*}/N}$$

where $x_{i}^{\prime }$ represent voxels of the resampled and intensity-matched reconstructed volume. We also give a value of peak signal to noise ratio (PSNR) which can be expressed as

(12) $$\mathrm{PSNR}(X^{\prime }\text{,}X^{*})=20\log_{10}\left(\frac{\mathrm{MAX}}{\sqrt{\sum_{i}{\left(x_{i}^{\prime }-x_{i}^{*}\right)}^{2}/N}}\right)$$

where *MAX* was set to maximum value of the original neonatal image.

Secondly, we also calculated the target registration error (TRE) between the simulated and the estimated transformations. TRE is defined as

(13) $$\mathrm{TRE}(T^{\prime }\text{,}T^{*})=\frac{\sum_{\mathrm{jk}}(\text{dist}(T_{k}^{\prime }(u_{\mathrm{jk}})\text{,}T_{k}^{*}(u_{\mathrm{jk}}))}{\sum_{k}N_{k}}$$

where $u_{\mathrm{jk}}$ denote locations of voxels in the simulated slices. $T^{*}=\{T_{1}^{*}\text{,}\ldots \text{,}T_{K}^{*}\}$ denote the simulated transformations and transformations $T^{\prime }=\{T_{1}^{\prime }\text{,}\ldots \text{,}T_{K}^{\prime }\}$ refer to recovered alignment of the slices to the reconstructed volume, composed with the transformation of the reconstructed volume to the original volume, as estimated using rigid registration implemented in IRTK. Operator dist(.,.) refers to Euclidean distance. The TRE is averaged over all voxels in the slices located within the region of interest, which in the case of neonatal data is obtained by excluding the dark background. To obtain a sensible TRE in the experiments below, we excluded slices which were not expected to be correctly aligned, but were instead expected to be excluded during the reconstruction, as the quality of their alignment does not affect the quality of the reconstruction. The criteria for exclusion were: (1) the slice has been corrupted during simulation; (2) during simulation the slice has been assigned a large displacement outside the capture range of the registration method; (3) the slice cannot be correctly registered due to a small region of interest. Slices falling into any of these tree categories were excluded from further calculations, and therefore the TRE presented below only reflects the quality of alignment of the slices contributing to the reconstructed volume.

Quantitative evaluation of the six simulated datasets is shown in Tables 3 and 4. The average NMRSE, PSNR (Table 3) and TRE (Table 4) are shown for the three 3-stack reconstructions (column 1), the three 6-stack reconstructions (column 2) and all six reconstructions (column 3). In column 4 we present the *p*-value to evaluate whether the performance of each method is significantly different to our proposed method. The *p*-value was calculated using paired Student’s *t*-Test with two-tailed distribution. In all cases, the proposed method performed better than the method with Huber statistics. Exclusion of either robust statistics or intensity matching resulted in a substantial drop in performance. All these differences in performance were statistically significant (*p*-value 0.05). The results achieved using the proposed method were similar (not significantly different for *p*-value 0.05) to the reference reconstruction, in which no scaling factors or bias fields were applied to simulated slices and no intensity matching was applied during reconstruction. Thus we can conclude that intensity matching successfully removes inconsistencies in bias fields and scaling factors. The quantitative results also show that in the presence of motion artifacts, using six stacks results in better registration accuracy and reconstruction results compared to using only three stacks.

**Fig. 5:**
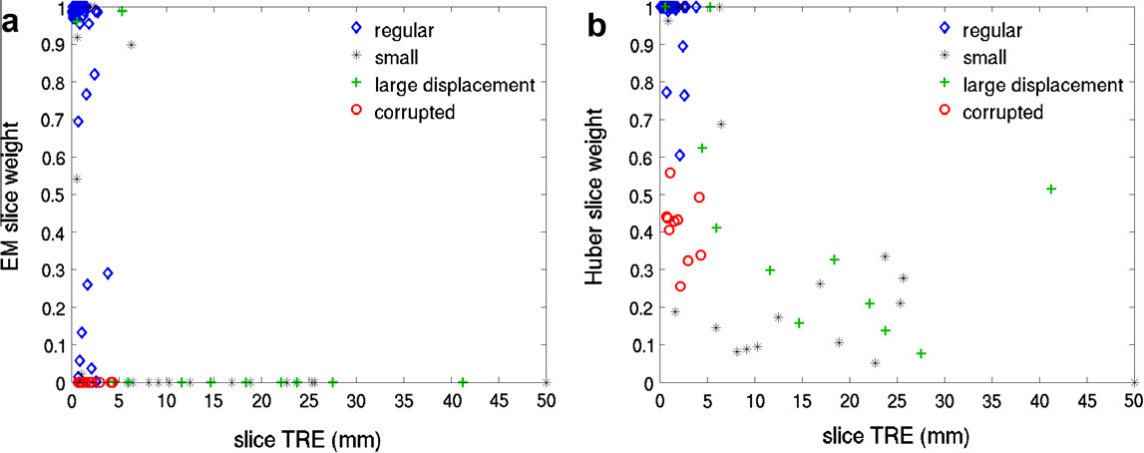
Comparison of slice weights using (a) EM and (b) Huber robust statistics, plotted against TRE calculated for each slice. Corrupted slices are shown as red circles and the slices which have been deliberately assigned large displacements during simulation are denoted by green crosses. Black asterisks denote slices with small region of interest with little information to guide registration towards correct alignment. The corrupted and misplaced slices are completely removed using EM robust statistics (zero weights), while their weight is only reduced when Huber robust statistics are used. (For interpretation of the references to color in this figure legend, the reader is referred to the web version of this article.)

Fig. 5 compares slice weights assigned by EM and Huber robust statistics for reconstruction from six simulated stacks. It can be seen that EM robust statistics assign zero weights to all corrupted and obviously misregistered slices, while Huber statistics only reduce their weights. Several slices have rather small TRE, but were assigned relatively small weights (below 0.5) by EM robust statistics. Visual inspection revealed that these are slices with high information content, where a small displacement can result in a relatively large intensity error. Conversely, some slices with small regions of interest and low information content, which are especially prone to misregistrations, can exhibit rather small intensity errors despite larger TRE and are thus not excluded.

### 4.2 Reconstruction of clinical fetal data

We have applied our reconstruction method to 10 clinical fetal brain MRI with gestational ages (GA) 20, 21, 23, 25 (2 cases), 26, 27, 33 and 37 weeks (see Table 5). The data were acquired at John Radcliffe Hospital, Oxford, on a Philips Achieva 1.5T scanner with a dedicated body coil, using a T2 weighted single shot turbo spin echo sequence with parameters TR = 32805 ms, TE = 100 ms, flip angle 90°. The slice thickness was 3 or 4 mm with a slice gap of 0.5 mm. The slices were acquired with in-plane resolution 1.235mm×2.276mm and automatically resampled on the scanner to resolution 0.75×0.75mm. The scanning time was 33 s per sequence. Several stacks were acquired in each of the three orthogonal directions, usually between 1 and 3, with 4–12 stacks overall. As only resampled slices were available, the FWHM of the PSF (modeled as a 3D Gaussian) could only be correctly set in through-plane direction, equal to the slice thickness. For the in-plane direction the FWHM was simply set to the voxel dimension of the resampled slice (0.75 mm).

**Fig. 6:**
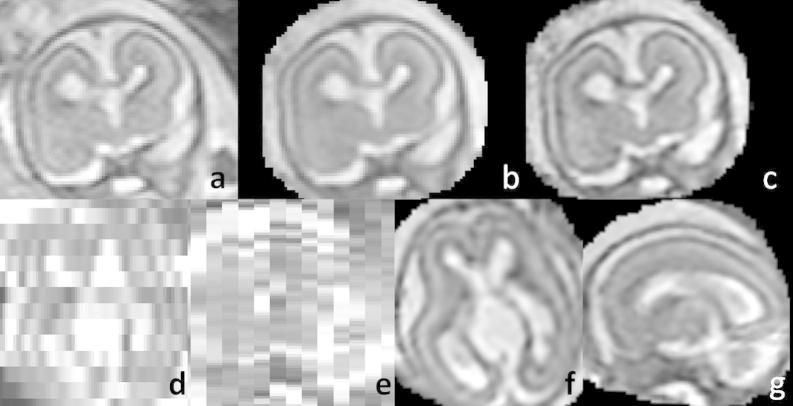
Brain MRI of a 20 week old fetus reconstructed from only 64 slices of 4 mm thickness using the proposed method. First row: (a) An acquired slice. (b) The same slice simulated from the reconstructed volume. (c) Corresponding plane from the reconstructed volume. Second row: Axial and transversal planes orthogonal to the acquired slice shown in (a): original stacks (d and e) and the reconstructed volume (f and g).

**Fig. 7:**
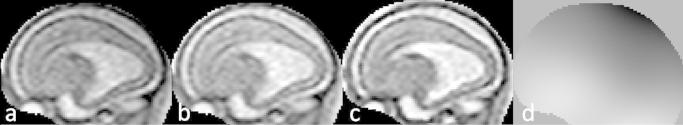
An example of the bias field estimated during reconstruction of the 23 week old fetus: (a) An acquired slice. (b) The scaled and bias-corrected slice. (c) The corresponding plane of the reconstructed volume. (d) The estimated bias field.

**Fig. 8:**
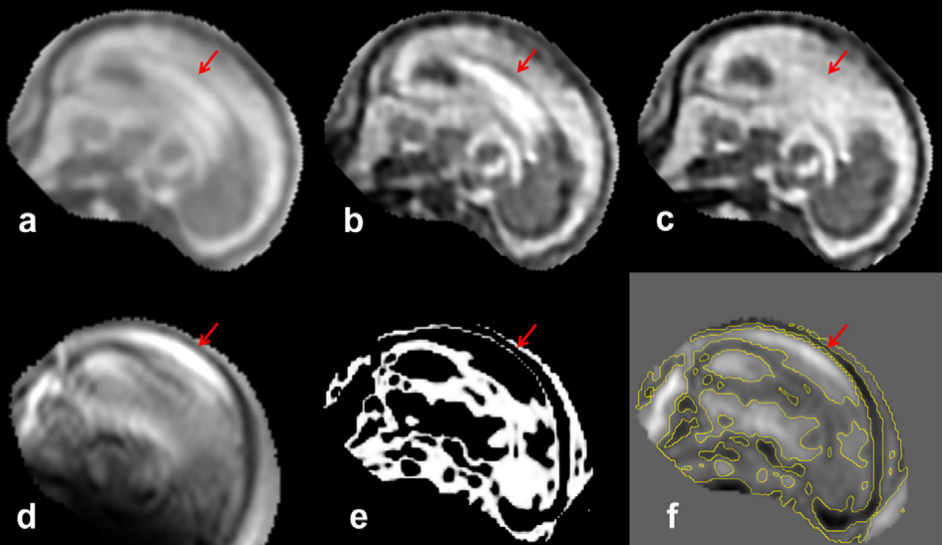
Artifacts in the reconstructed volume of the 23 weeks old fetus caused by a corrupted slice. (a) Initialization using Gaussian weighted reconstruction (Rousseau et al., 2006). (b) Reconstruction without robust statistics. (c) Reconstruction with EM robust statistics. (d) The corrupted slice. (e) The posteriors $p_{\mathrm{jk}}$. (h) The error between the corrupted slice and the corresponding simulated slice with an overlaid 0.5 isoline of the posteriors. The red arrow points to the skull in the misaligned corrupted slice, which appears as an artifact in the reconstructions if no robust statistics are used. (For interpretation of the references to color in this figure legend, the reader is referred to the web version of this article.)

**Fig. 9:**
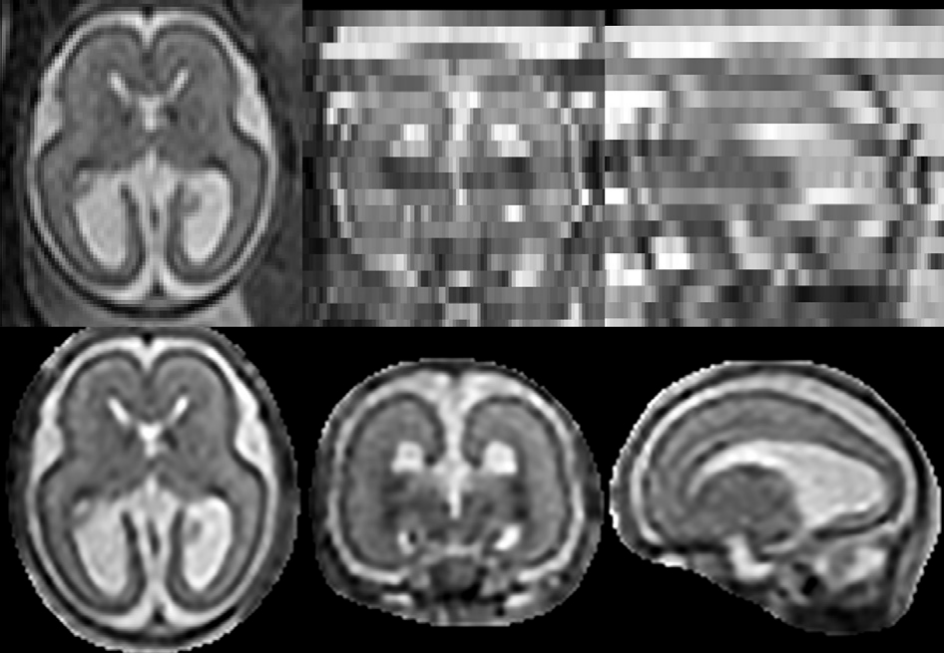
Reconstructed fetal brain MRI of a subject with GA 23 weeks using only five stacks of slices. First row: One of the acquired stacks. Second row: Reconstructed volume.

**Fig. 10:**
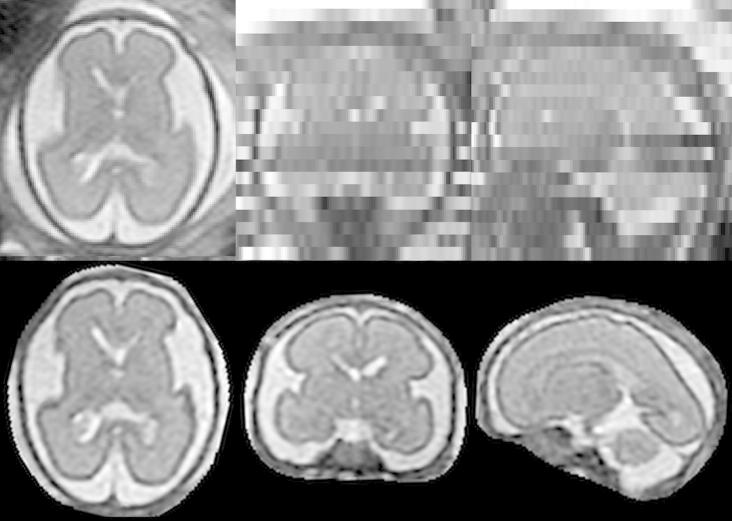
Reconstructed fetal brain MRI of a subject with GA 26 weeks using only five stacks of slices. First row: One of the acquired stacks. Second row: Reconstructed volume.

**Fig. 11:**
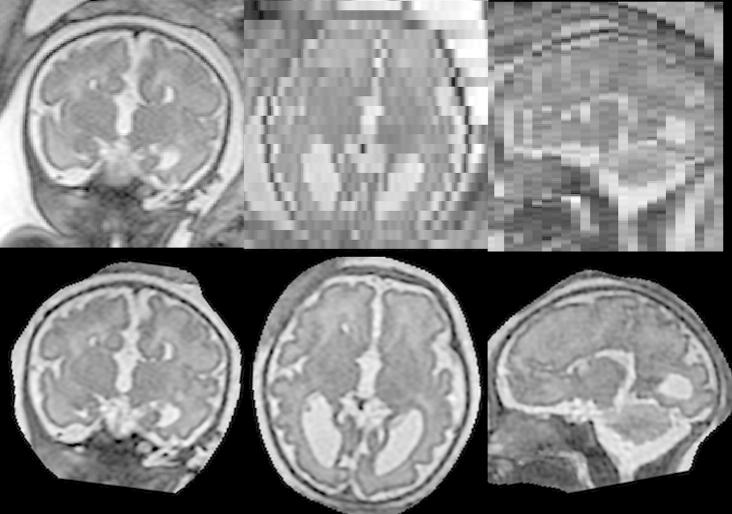
Reconstructed fetal brain MRI of a subject with GA 34 weeks using only five stacks of slices. First row: One of the acquired stacks. Second row: Reconstructed volume.

The reconstructed data were subjected to detailed visual inspection. We were able to achieve high-quality results for all 10 datasets. The proposed method is capable of reconstruction of very challenging datasets, such as the subject with 20 weeks GA for which only 64 slices with thickness 4 mm were available (see Fig. 6). Fig. 7 shows an example of the estimated bias field. Fig. 8 demonstrates the effectiveness the EM robust statistics in dealing with corrupted or misaligned slices. Fig. 8a shows the initialization of the reconstruction with obvious artifacts caused by a corrupted slice (Fig. 8d). If no robust statistics are used, the artifacts are still present in the final reconstruction (Fig. 8b). These are successfully removed using EM robust statistics (Fig. 8c). The posteriors and the errors for this slice are shown in Fig. 8e and f. Figs. 9–11 present the reconstructed volumes of the subjects with GA 23, 26 and 34 weeks, respectively.

### 4.3 Leave-one-out analysis of clinical data reconstruction

**Fig. 12:**
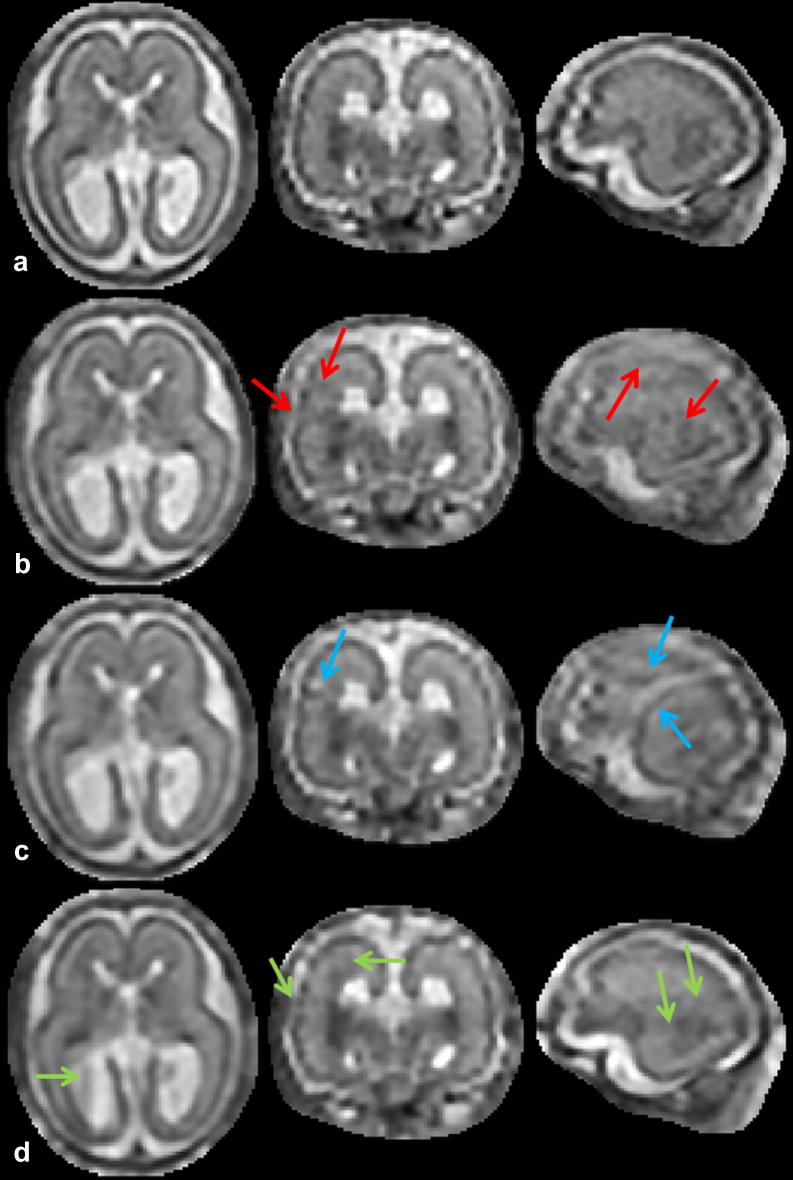
Reconstructions of the 23 week old fetus from four stacks using four methods evaluated in Table 6: (a) Reconstruction with EM robust statistics and intensity matching (Full). (b) Reconstruction with Huber robust statistics and intensity matching (Huber). (c) Reconstruction with no robust statistics and intensity matching (No robust). (d) Reconstruction with EM robust statistics and no intensity matching (No matching). Red arrows in column (b) show artifacts caused by incomplete removal of misregistered or corrupted slices when Huber robust statistics are used. Blue arrows in column (c) point to artifacts caused by misregistered or corrupted slices when no robust statistics is used. Green arrows show artifacts of intensity inconsistencies in column (d). Compare to Fig. 9 where all five stacks of the same dataset were used for reconstruction using our method. (For interpretation of the references to color in this figure legend, the reader is referred to the web version of this article.)

In previous studies, quantitative evaluation of fetal reconstruction methods was limited to simulated experiments, as there is no ground truth volume available for the real fetal data. In this paper we propose a new way of quantitatively evaluating reconstruction of the real data using leave-one-out strategy. In the absence of volumetric data, we can measure how well the reconstructed volume matches a stack of slices, which has been excluded from the reconstruction, by aligning these slices to the volume and simulating the acquisition. We can then perform a quantitative test by comparing these simulated slices the acquired slices in the evaluation stack. For each of the 10 clinical datasets, one stack was chosen for evaluation and another three to five stacks were chosen to reconstruct the volumetric image, depending on availability. The evaluation stacks were checked for corrupted slices and registered to the reconstructed volume, followed by iterating between slice-to-volume registration and intensity-matching proposed in this paper. The process converged when the sum-of-squared-differences between slices of the evaluation stacks and corresponding slices simulated from the reconstructed stack achieved no further improvement. After convergence, the NRMSE was calculated similarly to Eq. (11), except that averaging was performed over the voxels of the evaluation stack and normalized by the average intensity of the reconstructed volume. Table 6 shows quantitative results of this leave-one-out evaluation using the first four methods summarized in Table 2. Exclusion of either intensity matching or robust statistics results in drop of performance compared to the full method proposed in this paper, which is statistically significant (*p*-value 0.05), as shown by the third column of Table 6. The proposed method also achieves a statistically significant improvement compared to the method with Huber statistics, while Huber statistics still performs better compared to using no robust statistics. In Fig. 12 we present the reconstruction results of the leave-one-out analysis using the four methods quantitatively evaluated in Table 6. We can observe artifacts of misregistration (Fig. 12c) and intensity inconsistencies (Fig. 12d) if no robust statistics or no intensity matching are applied during reconstruction. Fig. 12b demonstrates artifacts of incomplete removal of misregistered or corrupted slices using the Huber function, which may also negatively influence the registration accuracy. None of these artifacts are present when both intensity matching and EM robust statistics are used (Fig. 12a). Nevertheless, we can still observe discontinuities in the coronal and sagittal view of Fig. 12a. These are caused by an insufficient number of slices used for reconstruction and consequently an incomplete coverage of the volume. The reconstruction quality can be enhanced by providing additional data (compare with Fig. 9).

### 4.4 Sufficient number of stacks

When deciding on a scanning protocol for acquiring fetal brain MRI, it is important to understand how many stacks of slices are needed so that unnecessary stress on the patient is not imposed. Additionally, increasing the number of stacks demands more computing resources in terms of memory and computational time. One of the most demanding parts of the algorithm is the computation of matrices $M_{k}$, which consists of discretized oriented 3D Gaussians (PSF). As calculating $M_{k}$ on the fly is too time-consuming, we only calculate it once for each motion-correction iteration and store it throughout super-resolution iterations. The amount of RAM needed for storage of $M_{k}$ can be quite large, especially for older subjects. Even when $M_{k}$ were stored, computational times for the experiments described in this paper were in the range of several hours. In our experiments we observed that in presence of motion artifacts, five stacks acquired by the clinical protocol described at the beginning of Section 4.2 are generally sufficient to produce good results. Reconstructed volumes presented in Figs. 6, 9, 10 and 11 were all obtained using five stacks. We performed an experiment with the subject with GA 27 weeks, for which 10 good quality stacks were available. The leave-one-out analysis showed that performance did not improve significantly for more than five stacks, though some marginal improvement was still achieved with nine stacks. Similar results were obtained for the subject with GA 33w. Even though these results are just preliminary, they can serve as a guidance when deciding on the scanning protocol for fetal brain MRI.

### 4.5 Reconstruction of data acquired using optimized sequences

**Fig. 13:**
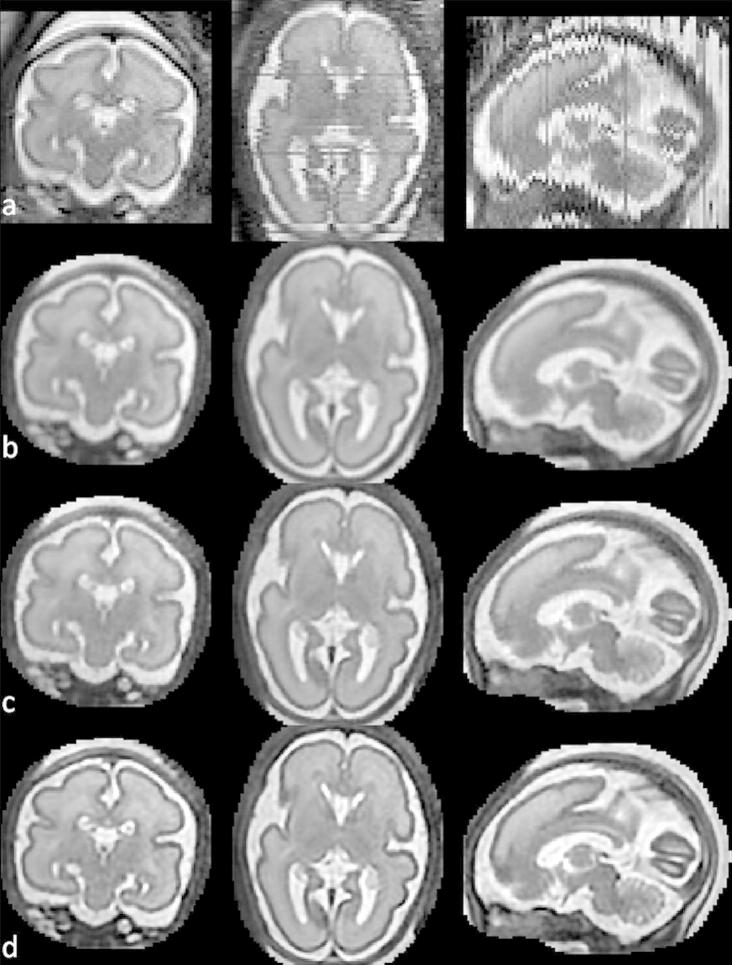
Reconstructed fetal brain MRI of a subject with GA 28 weeks using eight stacks of data acquired using optimized sequences. (a) One of the acquired stacks. (b) Reconstruction using multilevel B-splines. (c) Super-resolution reconstruction with PSF derived from the voxel spacing (FWHM 1.176 mm in-plane and 1.25 mm through-plane). (d) Super-resolution reconstruction with PSF matched to the data (FWHM 1.4 mm in-plane and 2.5 mm through-plane). The same motion correction parameters, determined by application of the full algorithm, were employed in all cases, with only the final reconstruction being performed using the three different reconstruction approaches. Intensity matching and robust statistics were used during all three reconstructions.

Previous methods tried to produce high-resolution reconstructions of fetal brain MRI in one of the two ways: Jiang et al. (2007) used thin slices and dense oversampling of the space followed by B-spline interpolation, while Gholipour et al. (2010) and Rousseau et al. (2010) used super-resolution techniques to reconstruct clinical thick slice data. In this section we investigate whether super-resolution techniques can also improve reconstruction of densely sampled thin slice data. We reconstructed three thin-slice datasets with GA 23, 28 and 36 weeks, acquired at Hammersmith Hospital, London (see Fig. 13a). The images were acquired on Philips Achieva 1.5T scanner with parameters TR = 15000, TE = 140–180 and excitation pulse of 90°. The datasets consist of eight stacks of thin slices, with in-plane resolution 1.176 mm, slice thickness 2.5 mm and slice overlap 1.25 mm. The sequences were designed to acquire approximately isotropic data. The PSF in-plane is a sinc function in both direction, and most of the signal is contained within four in-plane resolution units. This is approximately matched by a Gaussian with FWHM 2.5 mm which is a good approximation of the slice profile. The slice overlap of 50% of the slice thickness was chosen to ensure dense samples even when there is no movement.

The approximately isotropic nature makes these data suitable for reconstruction using multilevel B-splines (Lee et al., 1997). We reconstructed these datasets with multilevel B-splines combined with intensity matching and a simplified version of robust statistics (we exclude slices with weights smaller than 0.5) and this method produced good results (see Fig. 13b). To investigate whether super-resolution techniques could further improve the results, we also reconstructed these datasets using our proposed method. In the first experiment the PSF was set to match the resolution of the stacks with FWHM matched to the voxel size in each direction (see Fig. 13c). In the second experiment the PSF was chosen to closely approximate the real acquisition of the data. FWHM of the 3D Gaussian in-plane was set to FWHM of the central lobe of the sinc function (1.2 × in-plane resolution), and in through-plane direction to the slice thickness. The results shown in Fig. 13d suggest that the super-resolution reconstruction with the PSF correctly matched to the data can produce high-definition and high-resolution fetal brain volumes. We can observe that the definition of the structures is improved using the super-resolution technique compared to B-spline interpolation. The best results are achieved when the PSF reflects the true acquisition properties of the data, producing images with the best contrast-to-noise ratio.

## 5 Discussion

In this paper we described an algorithm which is designed to deal with the major artifacts in fetal brain MRI. We proposed a practical solution, which produced excellent results which can be used for further image analysis. We combined techniques best suited for the task in our opinion, sometimes at the expense of more rigorous theoretical modeling, e.g. by choosing different objective functions for reconstruction and motion correction. The development of a more rigorous mathematical framework, which would theoretically unify all elements in a single objective function, therefore remains an open problem at present.

We proposed to use a multi-resolution approach which makes use of temporal relaxation of the regularization requirement. Thus at the beginning of the process, when slices are not well aligned, the influence of a single slice is decreased, preventing registration of the slice against itself. We found this approach to be very effective especially in cases where a large amount of motion is present and only a small amount of data is available, often the case when reconstructing MRI of young fetuses. Such data are prone to getting trapped in a sub-optimal solution, when a misaligned slice is visible in the reconstructed volume rather than being correctly aligned. This happens largely due to the use of a redescending influence function to perform robust statistics which makes the convergence to the global optima not guaranteed. Thus the main goal of using a multi-resolution approach is to encourage the motion correction to find the global optima. If the amount of motion is small and more slices are available for reconstruction, the influence of the multi-resolution scheme is small and comparable results are obtained with and without temporally adaptive smoothing. We believe this is because the algorithm is initialised close to the global optimum, and a larger amount of data means that the global optimum is better defined.

In our preliminary experiments we estimated that five stacks of thick-slice clinical data (3–4 mm thick) with a gap (0.5 mm) are sufficient for good reconstruction of the fetal brain volume which is suitable for further analysis. Indeed, increasing the number of stacks may result in further improvement. In their work, Rousseau et al. (2010) established that under ideal conditions, three orthogonal stacks of thick slices with no gaps are sufficient if super-resolution reconstruction is combined with edge-preserving regularisation. They performed a reconstruction of fetal MRI of a sedated fetus demonstrating good results. However, up to nine stacks were needed when Gaussian noise was added. Gholipour et al. (2010) determined that nine stacks were needed to maximize the performance of the reconstruction, even though good reconstructions could be achieved with smaller number of stacks. As our clinical data contain gaps and motion-corrupted slices (and no sedation was used), it is natural that more than three stacks are needed to obtain good coverage of the volume. Our preliminary results are therefore in line with findings of the previous works.

## 6 Conclusion

In this paper we proposed a comprehensive methodology for reconstruction of fetal brain MRI. The method can deal with data with both large and small slice thickness as well as motion corruption and intensity artifacts present in fetal brain MRI. We described a particular implementation of the proposed framework, which produces excellent results. Moreover, the framework is flexible and different elements, such as the similarity measure for motion correction, robust statistics and the volume reconstruction can be changed or further developed. We proposed a novel robust statistics which completely removes identified outliers and thus enables us to reconstruct the volume from a small number of stacks. We also showed that proposed intensity matching is essential to produce artifact-free reconstructions. The method produced excellent results for clinical data, which are often acquired by protocols aimed at short scanning times and visually plausible images but are not optimal for the 3D reconstruction purposes. Thus the method is widely applicable, including situations when optimization of sequences is not possible or the data have already been acquired for other purposes. When our proposed methodology was applied to optimized densely sampled thin-sliced data, we achieved high-definition reconstructions, suitable for further image analysis and detailed quantification fetal brain development.

## Figures and Tables

**Table 1:** Six simulations used for evaluation. For each simulation we show number of stacks (second column) and average motion calculated as the average distance of a voxel in a simulated slice to its original location in the volume $X^{*}$ (third column). The fourth column gives the average voxel-wise magnitude of the bias field in all stacks, where bias fields in each slice have zero mean. The numbers in brackets give maximum slice-dependent average bias magnitude.

	Stacks	Motion (mm)	Bias magnitude
Simulation 1	3	1.34	0.07 (0.09)
Simulation 2	3	1.50	0.03 (0.05)
Simulation 3	3	2.38	0.06 (0.12)
Simulation 4	6	1.43	0.07 (0.09)
Simulation 5	6	1.84	0.05 (0.09)
Simulation 6	6	1.92	0.06 (0.12)

**Table 2:** Methods used to reconstruct simulated MR stacks for quantitative evaluation.

Method	Robust statistics	Intensity correction
Full	EM	Yes
Huber	Huber	Yes
No robust	No	Yes
No matching	EM	No
Reference	EM	No

**Table 3:** NRMSE and PSNR (in brackets) between image reconstructed from simulated stacks and original volume, averaged over simulations from 3 stacks, 6 stacks and all simulations. Last column presents *p*-value for NRMSE of all simulations, showing statistical significance of performance compared to the full method.

	3 Stacks	6 Stacks	All	*p*-Value
Full	0.112(26)	0.082(29)	0.097(27)	
Huber	0.123(25)	0.091(28)	0.107(27)	0.004
No robust	0.160(23)	0.126(25)	0.143(24)	0.001
No matching	0.150(24)	0.111(26)	0.131(25)	0.002
Reference	0.119(25)	0.085(28)	0.102(27)	0.206

**Table 4:** TRE between known and recovered alignment of the simulated slices, averaged over simulations from 3 stacks, 6 stacks and all simulations. Last column presents *p*-value for all simulations, showing statistical significance of performance compared to the full method.

	3 Stacks (mm)	6 Stacks (mm)	All (mm)	*p*-Value
Full	0.83	0.72	0.77	
Huber	0.92	0.74	0.83	0.039
No robust	1.05	0.80	0.95	0.001
No matching	0.96	0.78	0.87	0.015
Reference	0.76	0.65	0.72	0.160

**Table 5:** Ten clinical datasets. We show the gestational age of the fetus, the number of good quality stacks used for the reconstruction, the number of the slices after brainmasking, the number of the slices included in the reconstruction after exclusion of corrupted and misregistered slices using EM robust statistics and slice thickness.

GA	Stacks	Slices	Included slices	Slice thickness (mm)
20w	5	72	64	4
21w	5	62	49	4
23w	5	79	60	3–4
25w	4	62	47	4
25w	7	140	120	4
26w	5	92	78	3–4
27w	5	112	96	3–4
33w	5	130	120	3
34w	5	121	115	4
37w	6	154	141	4

**Table 6:** Average NRMSE obtained from leave-one-out evaluation of 10 clinical datasets. Last column shows *p*-value, showing statistical significance of performance compared to the full method.

Method	NRMSE	*p*-Value
Full	0.117±0.011	
Huber	0.120±0.012	0.02
No robust	0.125±0.015	0.03
No matching	0.129±0.024	0.02

## Footnotes

2 Image Registration Toolkit (IRTK), www.doc.ic.ac.uk/∼dr/software.

## References

[b0005] Ashburner J., Friston K.J. (2005). Unified segmentation. NeuroImage.

[b0010] Bertelsen, A., Aljabar, P., Xue, H., Srinivasan, L., Hayat, T., Allsop, J., Rueckert, D., Rutherford, M.R., Hajnal, J.V., 2009. Improved slice to volume reconstruction of the fetal brain for automated cortex segmentation. In: Proceedings of the International Society for, Magnetic Resonance in Medicine, p. 3437.

[b0015] Charbonnier P., Blanc-Feraud L., Aubert G., Barlaud M. (1997). Deterministic edge-preserving regularization in computed imaging. IEEE Transactions on Image Processing.

[b0020] Clouchoux C., Kudelski D., Gholipour A., Warfield S., Viseur S., Bouyssi-Kobar M., Mari J.L., Evans A., du Plessis A., Limperopoulos C. (2012). Quantitative in vivo MRI measurement of cortical development in the fetus. Brain Structure and Function.

[b0025] Damodaram M., Story L., Allsop J., McGuinness A., Patel A., Kumar S., Rutherford M. (2009). 3-dimensional MR reconstruction and brain volumetry in IUGR fetuses. International Journal of Gynecology and Obstetrics.

[b0030] Damodaram M., Story L., Allsop J., McGuinness A., Patel A., Kumar S., Rutherford M. (2009). Three-dimensional MR reconstruction and evaluation of the cerebellum to whole brain ratio in IUGR fetuses. International Journal of Gynecology and Obstetrics.

[b0035] Dubois J., Benders M., Borradori-Tolsa C., Cachia A., Lazeyras F., Ha-Vinh Leuchter R., Sizonenko S.V., Warfield S.K., Mangin J.F., Huppi P.S. (2008). Primary cortical folding in the human newborn: an early marker of later functional development. Brain.

[b0040] Duda R., Hart P., Stork D. (2001). Pattern Classification.

[b0045] Gholipour A., Estroff J., Barnewolt C., Connolly S., Warfield S. (2011). Fetal brain volumetry through MRI volumetric reconstruction and segmentation. International Journal of Computer Assisted Radiology and Surgery.

[b0050] Gholipour A., Estroff J., Warfield S. (2010). Robust super-resolution volume reconstruction from slice acquisitions: application to fetal brain MRI. IEEE Transactions on Medical Imaging.

[b0055] Gholipour, A., Warfield, S.K., 2009. Super-resolution reconstruction of fetal brain MRI. In: MICCAI Workshop on Image Analysis for the Developing Brain (IADB).

[b0060] Greenspan H. (2009). Super-resolution in medical imaging. Comput. J..

[b0065] Habas P., Scott J., Roosta A., Rajagopalan V., Kim K., Rousseau F., Barkovich J., Glenn O., Studholme C. (2012). Early folding patterns and asymmetries of the normal human brain detected from in utero MRI. Cerebral Cortex.

[b0070] Jiang S., Xue H., Glover A., Rutherford M., Rueckert D., Hajnal J. (2007). MRI of moving subjects using multislice snapshot images with volume reconstruction (SVR): application to fetal, neonatal, and adult brain studies. IEEE Transactions on Medical Imaging.

[b0075] Kim K., Habas P., Rajagopalan V., Scott J., Corbett-Detig J., Rousseau F., Barkovich A., Glenn O., Studholme C. (2011). Bias field inconsistency correction of motion-scattered multislice MRI for improved 3D image reconstruction. IEEE Transactions on Medical Imaging.

[b0080] Kim, K., Habas, P., Rajagopalan, V., Scott, J., Rousseau, F., Barkovich, A.J., Glenn, O., Studholme, C., 2011b. Robust 3D reconstruction from motion scattered multislice MRI using second order models and structure tensor weighted kernel regression. In: MICCAI Workshop on Image Analysis of Human Brain Development, Toronto, Canada.

[b0085] Kim K., Habas P., Rousseau F., Glenn O., Barkovich A., Studholme C. (2010). Intersection based motion correction of multislice MRI for 3-D in utero fetal brain image formation. IEEE Transactions on Medical Imaging.

[b0090] Lee S., Wolberg G., Shin S. (1997). Scattered data interpolation with multilevel b-splines. IEEE Transactions on Visualization and Computer Graphics.

[b0095] Leemput K.V., Maes F., Vandermeulen D., Suetens P. (1999). Automated model-based bias field correction of MR images of the brain. IEEE Transactions on Medical Imaging.

[b0100] Limperopoulos C., Clouchoux C. (2009). Advancing fetal brain MRI: targets for the future. Seminars in Perinatology.

[b0105] Milanfar P. (2007). Super-Resolution Imaging.

[b0110] Ohbuchi, R., Chen, D., Fuchs, H., 1992. Incremental volume reconstruction and rendering for 3D ultrasound imaging. In: SPIE Visualization in Biomedical, Computing, pp. 312–323.

[b0115] Peyre G. (2011). A review of adaptive image representations. IEEE Journal of Selected Topics in Signal Processing.

[b0120] Prayer D., Brugger P.C., Prayer L. (2004). Fetal MRI: techniques and protocols. Pediatric Radiology.

[b0125] Rajagopalan V., Scott J., Habas P., Kim K., Corbett-Detig J., Rousseau F., Barkovich J., Glenn O., Studholme C. (2011). Local tissue growth patterns underlying normal fetal human brain gyrification quantified in utero. The Journal of Neuroscience.

[b0130] Rajagopalan V., Scott J., Habas P., Kim K., Rousseau F., Glenn O., Barkovich J., Studholme C. (2011). Spatiotemporal morphometry of adjacent tissue layers with application to the study of sulcal formation. Medical Image Computing and Computer Assisted Intervention.

[b0135] Rousseau F., Glenn O.A., Iordanova B., Rodriguez-Carranza C., Vigneron D.B., Barkovich J.A., Studholme C. (2006). Registration-based approach for reconstruction of high-resolution in utero fetal MR brain images. Academic Radiology.

[b0140] Rousseau F., Kim K., Studholme C., Koob M., Dietemann J.L. (2010). On super-resolution for fetal brain MRI. Medical Image Computing and Computer Assisted Intervention.

[b0145] Rousseau, F., Oubel, E., Pontabry, J., Studholme, C., Koob, M., Dietemann, J.L. 2011. An open-source toolkit for fetal brain MR image processing. In: MICCAI Workshop: Image Analysis of Human Brain Development.

[b0150] Rutherford M., Jiang S., Allsop J., Srinivasan L., Hayat T., Kumar S., Hajnal J. (2008). MR imaging methods for assessing fetal brain development. Developmental Neurobiology.

[b0155] Studholme C. (2011). Mapping fetal brain development in utero using magnetic resonance imaging: the big bang of brain mapping. Annual Review of Biomedical Engineering.

[b0160] Studholme C., Hills D., Hawkes D. (1999). An overlap invariant entropy measure of 3D medical image alignment. Pattern Recognition.

[b0165] Tolsa C.B., Zimine S., Warfield S.K., Freschi M., Rossignol A.S., Lazeyras F., Hanquinet S., Pfizenmiaer M., Huppi P.S. (2004). Early alteration of structural and functional brain development in premature infants born with intrauterine growth restriction. Pediatric Research.

[b0170] Wells W., Grimson W., Kikinis R., Jolesz F. (1996). Adaptive segmentation of MRI data. IEEE Transactions on Medical Imaging.

